# Multivariable analysis to determine if HIV-1 Tat dicysteine motif is associated with neurodevelopmental delay in HIV-infected children in Malawi

**DOI:** 10.1186/s12993-015-0083-7

**Published:** 2015-12-17

**Authors:** Jasmeen Dara, Anna Dow, Elizabeth Cromwell, Christa Buckheit Sturdevant, Macpherson Mallewa, Ronald Swanstrom, Annelies Van Rie, Vinayaka R. Prasad

**Affiliations:** Department of Pediatrics, Montefiore Medical Center, Bronx, NY USA; UNC School of Public Health, University of North Carolina, Chapel Hill, NC USA; Department of Biochemistry and Biophysics, University of North Carolina, Chapel Hill, NC USA; Malawi-Liverpool Wellcome Trust and Department of Pediatrics, College of Medicine, Blantyre, Malawi; Department of Microbiology and Immunology, Albert Einstein College of Medicine, Bronx, NY USA

**Keywords:** HIV-1, HIV-1 subtype C, Tat, Dicysteine motif, Encephalopathy, Neurodevelopment

## Abstract

**Background:**

HIV-1 Tat protein is implicated in HIV-neuropathogenesis. Tat C31S polymorphism (Tat^CS^) has been associated with milder neuropathology in vitro and in animal models but this has not been addressed in a cohort of HIV-infected adults or children.

**Methods:**

HIV viral load (VL) in plasma and cerebrospinal fluid (CSF) were determined and plasma HIV *tat* gene was sequenced. Neurodevelopmental assessment was performed using Bayley Scales of Infant Development III (BSID-III), with scores standardized to Malawian norms. The association between Tat^CS^ and BSID-III scores was evaluated using multivariate linear regression.

**Results:**

Neurodevelopmental assessment and HIV *tat* genotyping were available for 33 children. Mean age was 19.4 (SD 7.1) months, mean log VL was 5.9 copies/mL (SD 0.1) in plasma and 3.9 copies/mL (SD 0.9) in CSF. The prevalence of Tat^CC^ was 27 %. Z-scores for BSID-III subtests ranged from −1.3 to −3.9. Tat^CC^ was not associated with higher BSID-III z-scores.

**Conclusions:**

The hypothesis of milder neuropathology in individuals infected with HIV Tat^CS^ was not confirmed in this small cohort of Malawian children. Future studies of *tat* genotype and neurocognitive disorder should be performed using larger sample sizes and investigate if this finding is due to differences in HIV neuropathogenesis between children and adults.

## Background

Although prevention of mother-to-child transmission (PMTCT) of HIV has significantly decreased the incidence of pediatric HIV, approximately 240,000 children were newly infected with HIV in 2013 [1]. HIV infection negatively impacts the neurological development of children, with symptoms ranging from mild neurodevelopmental delays to severe neurocognitive and motor impairments [2–4]. While early initiation of anti-retroviral therapy (ART) can prevent or delay the onset of HIV-associated neurological disorders (HAND) [5, 6], only one in every seven children living with HIV in sub-Saharan Africa receive ART [7].

Virologic factors that contribute to neurodevelopment are poorly understood, particularly in children. HIV-1 Tat protein has been implicated in both directly [8] and indirectly [9] inducing monocyte chemotaxis, damaging the blood brain barrier, direct neurotoxicity and augmenting monocyte infiltration across the blood–brain barrier. Tat is released from infected cells and causes direct damage to the central nervous system (CNS) white matter [10, 11] and promotes indirect damage through the induction of chemokines and cytokines [12]. A key molecular determinant of neuropathogenesis in HIV-1 Tat is the C30C31 dicysteine motif (Tat^CC^) and in a given individual’s plasma, nearly all viruses display the same genotype. For example, Tat in HIV-1 subtype B isolates contains a highly conserved (99 %) C30C31 motif, which mediates its ability to induce β chemokines and promote monocyte chemotaxis [13]—features that play a key role in HAND. In contrast, approximately 90 % of subtype C HIV-1 isolates worldwide have a C31S (Tat^CS^) polymorphism [13], which has been linked with defective monocyte chemotaxis. We have previously shown that an Indian subtype C HIV-1 isolate with Tat^CS^, when compared to US subtype B isolate, displayed decreased in vitro chemokine induction, decreased monocyte recruitment by infected macrophages, and in a mouse model for HIV encephalitis [14], caused milder neurodegeneration and decreased cognitive impairment following intracranial injection of HIV-infected monocyte-derived macrophages [15]. Furthermore, subtype C HIV Tat^CS^ causes decreased N-methyl d-aspartate (NMDA) receptor induced neurotoxicity [16] and neuronal cell death [17] compared with subtype B HIV Tat.

The importance of Tat dicysteine motif in HAND is further underscored by the geographic variability of HIV-associated dementia (HAD) in adults in regions where subtype C predominates. In India, where subtype C infection is predominant, HAD prevalence of 2–6 % has been reported [18, 19]. In contrast, South Africa and Botswana, also countries with predominantly subtype C infections, have reported a 25–30 % prevalence of HAD [20, 21]. The higher incidence of HAD correlates with a higher prevalence of intact Tat dicysteine motif in Southern African isolates (11–22 % of subtype C HIV isolates from Botswana and South Africa), while the lower incidence of HAD in India correlates with a lower prevalence of HIV-1 isolates with Tat C30C31 motif [22]. Thus, the variation in the prevalence of HAND between different regions with subtype C HIV epidemics appears to correlate with regional differences in the frequency of the subtype C HIV-1 isolates bearing Tat with an intact dicysteine motif [22]. This hypothesis has been further validated through in vitro experiments and mouse models of HIV encephalitis using a Zambian HIV-1 subtype C isolate with an intact dicysteine motif [22]. However, the *tat* genotype and HAD prevalence in the same cohort has not been confirmed so far. A recent study in adults in South Africa showed no significant cognitive differences between adults with subtype C HIV-1 with TatC31S as compared to those with the dicysteine motif [23]. However, to date, viral genotypic differences related to pediatric HIV neurodevelopmental delay have not been evaluated.

We evaluated the relationship between the HIV-1 Tat dicysteine motif and neurodevelopmental delay in HIV-infected children in Malawi using the Bayley Scales of Infant Development III (BSID-III). We hypothesized that HIV-1 Tat dicysteine motif is associated with neurodevelopmental delay in children infected with HIV-1 subtype C.

## Methods

### Study population

Children were eligible to participate if HIV-infected, age 2–36 months, ART-naïve, but eligible for ART and if their primary caregiver had no chronic illnesses other than HIV that might impede the daily activities or neurodevelopment of the child. Children were recruited through either the pediatric HIV clinic or the emergency department at Queen Elizabeth Central Hospital in Blantyre, Malawi between June 2009 and June 2010. Children with a history of severe birth asphyxia, cerebral malaria, meningitis, a congenital malformation or chronic medical that may interfere with neurodevelopment were excluded.

Children recruited through the HIV clinic had to demonstrate delay for one or more neurodevelopmental milestones on a parental screening questionnaire, whereas those recruited through the emergency department had to have a clinical indication for a lumbar puncture.

### Laboratory and neurodevelopmental evaluations

Participating children underwent neurodevelopmental testing with the BSID-III, to assess motor (fine and gross), cognitive, and language (expressive and receptive communication) development [24]. Neurodevelopmental assessment was performed at enrolment for children recruited through the HIV clinic and at 1-month follow-up visit among children recruited through the emergency department, to ensure that the acute illness did not interfere with the neurodevelopmental assessment. The BSID-III was administered in the local language of Chichewa by a study pediatrician or nurse trained in administration of the BSID-III. Scores were interpreted using normative data from Malawi [25]. Neurological development for each child was classified as normal (between mean and ±1 SD), mild delay (between −1 and −2 SD), severely delayed (>−2 SD), above normal (between +1 and +2 SD) or advanced (>+2 SD above mean) for each of the five subtests.

Blood and cerebrospinal fluid (CSF) were collected and viral loads (VL) were determined using Abbott RealTime HIV-1 assay with a lower limit of quantitation of <320 copies/mL. Plasma HIV RNA was used to create cDNA through reverse transcription, and viral exon I *tat* sequences (nucleotides 1–214, corresponding to codons 1–70), which includes the codons for amino acids 30 and 31 were determined. The amino acid at Tat codon 31, predicted using the nucleotide sequence, was used in the statistical analysis.

### Statistical analysis

Using Stata 12.0 (College Station, TX), we created separate linear regression models to assess the association between the outcomes of fine motor, gross motor, cognitive, expressive communication and receptive communication z-scores with Tat^CC^ as the primary exposure variable of interest. Potential covariates included maternal ART use (dichotomous), exposure to PMTCT (dichotomous), breastfeeding >6 months of life (dichotomous), breastfeeding at enrollment (dichotomous), gender, log plasma VL (continuous), log CSF VL (continuous), CSF-to-plasma VL ratio (continuous), and maternal age (continuous). Frequency distributions were calculated for these covariates among subjects with Tat^CS^ and Tat^CC^. Covariates were examined for their relationship with neurodevelopmental z-scores using t-test with unequal variances, Mann–Whitney U-test, Pearson’s correlation, and Spearman’s correlation, as appropriate.

Separate multivariable models were created for each neurodevelopmental z-score using an a priori approach in which variables were chosen based on biological plausibility of confounding the relationship between exposure and outcome. In addition, covariates with missing values could not be included in the model as this lead to small effective sample sizes. Based on these criteria, the variables chosen to include were age, log plasma VL, and log CSF VL. To assess for normality of the linear regression model, the standardized residuals were plotted on a histogram and p–p plot. Outliers with a Cook’s distance greater than 4/n were excluded. Additional models using robust regression diagnostics were created by excluding outliers with Cook’s distance >1. The assumptions of equal variances and linearity were tested using a residuals-versus-fitted plot. Multicollinearity was tested by using variance inflation factors (VIF) with a cut-off of >10.

We also performed a sensitivity analysis to determine if the exclusion of the six subjects lacking Tat sequencing information affected the estimate of the association between exposure and outcome.

### Ethics statement

Institutional Review Board approval was received from the College of Medicine in Blantyre, Malawi, the University of North Carolina in Chapel Hill, NC, and Albert Einstein College of Medicine in Bronx, NY. Written informed consent was obtained from all parents or guardians.

## Results

### Characteristics of study population

**Fig. 1:**
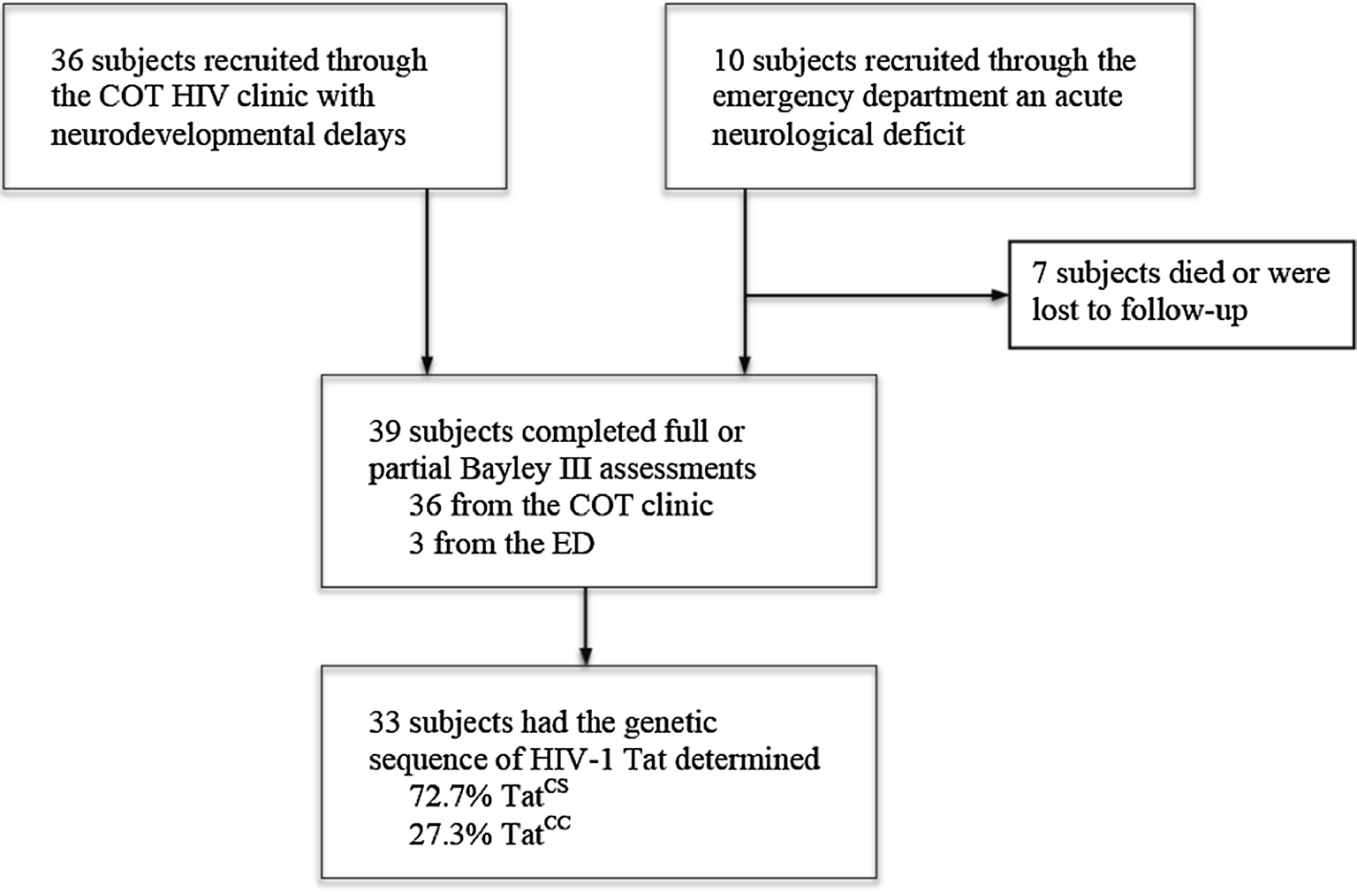
Study enrollment flow chart. Subjects were recruited either with neurodevelopmental delays through the cotrimoxazole (COT) HIV clinic or with acute neurological deficit requiring a lumbar puncture through the emergency department. Seven subjects died or were lost to follow-up. Thirty subjects completed full or partial Bayley III assessments and 33 of those subjects had isolates available for genetic sequencing

Of the 46 HIV-infected children enrolled, 39 completed neurodevelopmental assessments, and 33 had isolates available for genetic sequencing to determine the predicted amino acid sequence at Tat codon 31 (Fig. 1). Of the 33 children included in the analysis, 32 were recruited through the HIV clinic, and one through the emergency department. No HIV-negative children were enrolled. The mean age was 19.4 (SD 7.1) months, mean log plasma VL was 5.9 (SD 0.1) copies/mL, and mean log CSF VL was 3.9 (SD 0.9) copies/mL, and 44.8 % of all mothers in the study received single-dose nevirapine (sdNVP) for PMTCT. Compared to children with Tat^CS^, children with Tat^CC^ were more likely to have received sdNVP for PMTCT (p = 0.041), have higher log plasma VL (p = 0.008) and tended to have higher log CSF VL (p = 0.139) (Table 1). There was no difference in socioeconomic conditions such as ownership of goods, housing materials and access to running water between the two groups (data not shown).

**Table 1:** Demographic data

	Tat C31S polymorphism (Tat^CS^) n = 24 (72.7 %)	Dicysteine motif (Tat^CC^) n = 9 (27.3 %)	p-value
Age (months) mean ± SD*	20.2 ± 8.0	17.1 ± 7.1	0.261 t-test
Gender (% male)	45.8	44.4	>0.999 Fisher’s exact
Maternal age (years) mean ± SD	27.6 ± 4.8	26.7 ± 4.7	0.622 t-test, unequal
Log plasma viral load (copies/mL) mean ± SD	5.7 ± 0.6	6.4 ± 0.5	0.008 t-test, unequal
Log CSF viral load (copies/mL) mean ± SD	3.8 ± 1.0	4.3 ± 0.5	0.139 t-test
Viral load ratio (CSF:plasma) mean ± SD	0.04 ± 0.10	0.01 ± 0.01	0.401 t-test
Maternal ART^a^ use (% on ART)	19.1	0.0	0.552 Fisher’s exact
PMTCT^b^ (% that received sdNVP)	30.0	77.8	0.041 Fisher’s exact
Breastfeeding at enrollment (%)	30.4	25.0	>0.999 Fisher’s exact
Breastfed for >6months (%)	60.9	83.3	0.633 Fisher’s exact
Neurodevelopmental scores (z-scores)			t-test, unequal
Fine motor	−1.9 ± 1.1	−2.4 ± 1.4	0.361
Gross motor	−4.0 ± 1.7	−3.9 ± 1.3	0.783
Cognitive	−1.9 ± 1.1	−1.5 ± 0.8	0.199
Expressive communication	−1.9 ± 1.2	−1.9 ± 0.9	0.869
Receptive communication	−1.4 ± 0.9	−1.3 ± 0.7	0.916

* Sample size: age, gender, log viral load, log CSF viral load, viral load ratio, (n = 33); maternal age (n = 30); maternal ART use, PMTCT, breastfed for >6months (n = 29); breastfeeding at enrollment (n = 31)

^a^ART—antiretroviral therapy

^b^PMTCT—prevention of mother to child transmission; the use of antepartum antiretroviral therapy to prevent the peripartum transmission of HIV; all mothers that received PMTCT, received single-dose nevirapine (sdNVP)

### Exposure (HIV Tat motif) and outcome (neurodevelopment) in 33 Malawian children

The prevalence of the Tat^CC^ was 24 % among all (n = 41) pediatric isolates available and 27 % among those who also completed BSID-III evaluations (n = 33) (see Table 1).

**Fig. 2:**
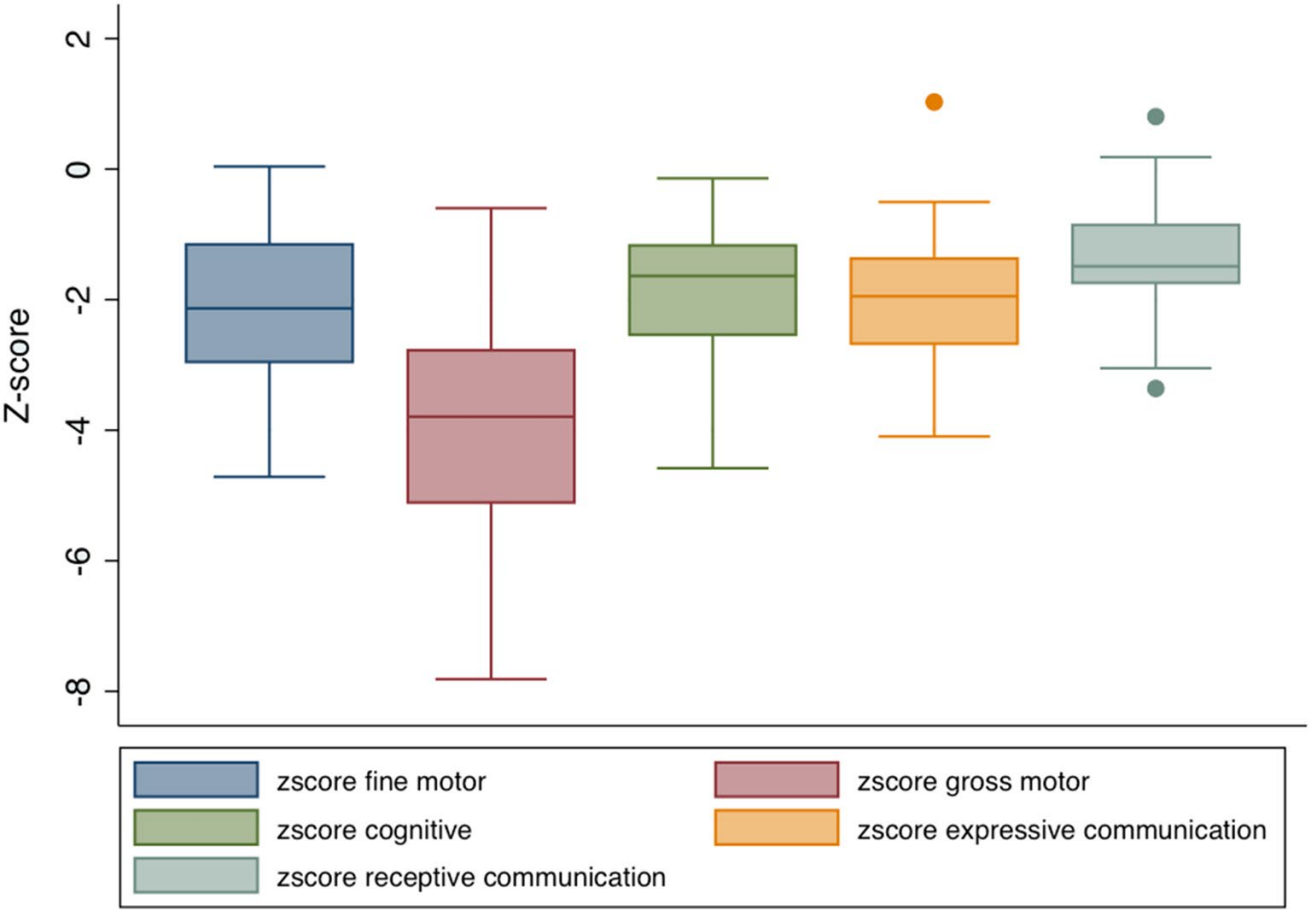
Neurodevelopmental z-scores obtained by the Bayley III Scales of Infant and Toddler Development standardized to pediatric Malawian norms. *Box* and *whiskers plot* mean and range for unadjusted neurodevelopmental z-scores, including gross motor, cognitive, expressive communication, and receptive communication

All children had moderate to severe delay in at least one domain: 88 % (n = 29) for cognitive development, 88 % (n = 29) for expressive communication, 60 % (n = 20) for receptive communication, 78 % (n = 26) for fine motor and 97 % (n = 32) for gross motor development (not shown). Unadjusted neurodevelopmental z-scores for each domain were plotted on a box and whiskers plot and shown in Fig. 2. Compared to the Malawian norm, the HIV infected children enrolled in the study had delayed development with z-scores on BSID-III ranging from −3.9 (SD 1.6) for gross motor development to −1.3 (SD 0.9) for receptive communication (Table 1; Fig. 2).

### Association between HIV Tat motif and outcome (neurodevelopment)

**Fig. 3:**
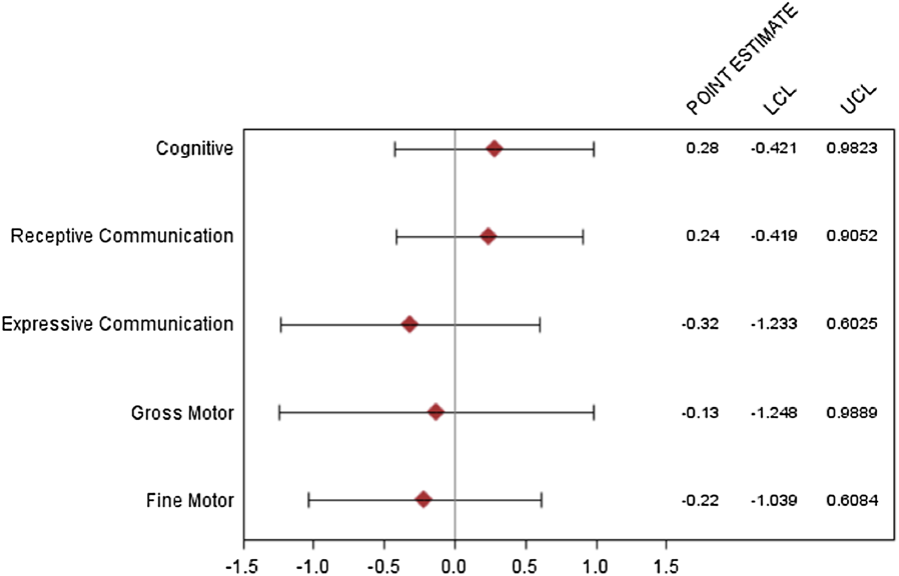
Adjusted coefficients for Tat^CC^. For each of the five subtests, the adjusted point estimate and 95 % lower confidence limit (LCL) and upper confidence limit (UCL) for the association for Tat^CC^ is plotted on the linear scale

The z-scores for the five subtests of the BSID-III did not differ between children infected with Tat^CC^ and Tat^CS^ HIV virus. In multivariate analyses, adjusting for age, log plasma VL, and log CSF VL in the final model, there was no significant association between Tat^CC^ and neurodevelopment, including fine motor (p = 0.314), gross motor (p = 0.732), cognitive (p = 0.553), expressive communication (p = 0.579) and receptive communication z-scores (p = 0.805) (Table 2). The results were similar when using robust regression analysis. Figure 3 illustrates the adjusted point estimates and 95 % confidence intervals for the association between Tat^CC^ and the five domains of neurological development measured by the BSID-III.

**Table 2:** P-values for Tat-CC in univariate and multivariable analysis

	Fine motor	Gross motor	Cognitive	Expressive communication	Receptive communication
Univariate analysis t-test, unequal variances	0.361	0.783	0.199	0.869	0.916
Multivariable analysis	0.846	0.974	0.155	0.848	0.376
Multivariable analysis excluding Cooks > 4/n	0.314	0.732	0.553	0.579	0.805
Multivariable analysis with robust regression	0.715	0.949	0.222	0.909	0.323

Adjusted for age, log plasma VL, and log CSF VL

Children excluded because of lack of Tat sequencing information were on average 5 months younger, and had higher gross motor and expressive communication z-scores compared to children with Tat information. In a sensitivity analysis to assess a potential bias introduced by the exclusion of these children, we found that assigning children with missing Tat information either as all Tat^CC^ or as all Tat^CS^ did not change our final result of a lack of association observed between Tat sequence and neurodevelopmental delay.

## Discussion

In this cross-sectional study of HIV-infected children in Malawi, we found, contradictory to our hypothesis, the presence of Tat^CC^ variants was not significantly associated with neurodevelopmental severity in multivariate analysis. This may be related to (1) differences in HIV-associated neuropathogenesis between children and adults, (2) small sample size or other factors.

It is possible that Tat^CC^ variants are associated with the occurrence of HIV neuropathogenesis but not the severity thereof, an issue we could not conclusively assess as all participating children presented with neurodevelopmental delay. Underlying differences in neuropathology may explain the results identified in this study. HIV neuropathy is a result of chronic HIV infection that results in the infiltration of activated monocytes into the brain, which leads to the production of Tat, gp120, cytokines, and other neurotoxic viral proteins [26]. In children with HIV-associated neuropathy, both the duration of HIV infection and the associated inflammation are shorter when compared with adults with HAND. Additionally, infants and children have rapidly developing brains, which undergo increases in white matter size, maturation of myelination, and increase in the number of neuronal axons [27]. Central nervous system (CNS) pathology of children with AIDS frequently involves global anoxic-ischemic and necrotizing encephalomyelopathies [28, 29] which differs from the pathology seen in adults consisting of fronto-temporal atrophy, diffuse parenchymal nodules, as well as perivascular mononuclear infiltrates [30]. Radiographic evaluations support white matter damage, which differs between neonatal and adult brains in response to ischemic injury [27]. Immunologic differences also distinguish pediatric and adult HIV-associated neuropathy. CNS vascular inflammation found in pediatric AIDS consists of transmural and perivascular mononuclear infiltrates consisting primarily of CD3+ and CD8+ T-lymphocytes, in contrast to the inflammatory lesions found in adult patients with HIV-1 encephalopathy in which monocytes and macrophages are the predominant mononuclear cells [31], suggesting the immunologic and inflammatory basis of disease in children is fundamentally different from that found in adults. A CNS compartmentalization study conducted on the same study population showed that only 10–20 % of the children had HIV variants compartmentalized in the CSF [32] that have evolved to use low levels of CD4, a characteristic of macrophage-tropic isolates. This supports the hypothesis that CNS HIV replication in the majority of the children occurs primarily in T-cells. Although prior in vitro research has shown that subtype C HIV-1 Tat with an intact dicysteine motif induces greater CCL-2 induction, monocyte recruitment, and neuropathology, the effects of HIV-1 Tat with an intact dicysteine motif on T-lymphocytes are unknown and merits evaluation. As previously noted, prior clinical data shows that Southern African countries with predominantly subtype C infection show both a higher prevalence of HAD and a higher proportion of subtype C HIV-1 with a Tat^CC^ motif [1, 21, 22, 33, 34]; however, all of these results were obtained from studies in adults. This is the first study to evaluate the neurodevelopmental outcomes in children infected with subtype C HIV-1 with the Tat^CC^ motif.

In the neurocognitive evaluation conducted by Paul et al. South African adult individuals infected with clade C HIV-1 with Tat^CC^ were compared to those infected with clade C HIV-1 with Tat^CS^ motif [23]. They found no differences in the neurocognitive defects between the two groups. This adult study, like the current pediatric study, also relied on plasma-derived HIV-1 sequences to determine the Tat genotype. It is known that HIV-1 can be compartmentalized in the brain [32] which could mean that it may be more directly relevant to correlate the cognitive defects to brain- or CSF-derived HIV-1 sequences than to plasma. Furthermore, studies focused on Tat also do not take into account the role of gp120, another source of neuronal dysfunction. Our recent report has shown that while the gp120 of Indian subtype C HIV-1 is non-neurovirulent, that of Southern African subtype C HIV-1 displayed robust neurovirulence [35].

Our findings should be interpreted in light of the study limitations. First, the sample size was small and information on some covariates was incomplete, which restricted our ability to adjust for confounding variables. Most importantly, even though in bivariate analysis we observed that children with Tat^CC^ were more likely to have higher plasma VL and were more likely to have received sdNVP for PMTCT (p = 0.041) compared to children with Tat^CS^, we were only able to include plasma VL and not PMTCT in the multivariate model. This may have biased the association to the null and thus contributed to the lack of a statistically significant relationship between HIV-1 Tat^CC^ motif and developmental delay. Second, data on the plasma CD4 or CD8 lymphocyte count was not available. Low plasma CD4 counts have been associated with neurocognitive dysfunction in children [36] and low plasma CD8 counts are associated with the risk of neurological impairment in children [37]. Third, the cross-sectional study design hindered the ability to infer causation and allowed for the introduction of biases. Survival bias may have occurred and influenced the interpretation of our findings. Although there may be some bias due to exclusion of children without Tat information, sensitivity analysis suggest that this did not influence our conclusions. In our analysis, children with HIV Tat^CC^ variants had a significantly higher mean plasma VL than HIV Tat^CS^ variants (p = 0.008; t-test, unequal variances). It is possible that HIV-infected children with Tat^CC^ variants did not survive as long as children with Tat^CS^ variants. If such survival bias occurred, it could appear that children with Tat^CS^ variants have more severe neurodevelopmental delay. Finally, we were unable to assess the association between Tat^CC^ and the severity of neurodevelopmental delay as all children included in the analysis presented with neurodevelopmental delay in at least one domain. A control group of HIV-infected children without neurodevelopmental delay could have contributed valuable information but was precluded given ethical and cultural concerns of performing a lumbar puncture in this population.

## Conclusions

In this study of 33 Malawian ART-naïve HIV-1 subtype C infected children with neurodevelopmental delay, we observed that the presence of HIV Tat^CC^ motif was not associated with the severity of developmental delay in children with HIV type C infection. This may be due to differences in HIV-1 neuropathogenesis in children as compared to adults but demonstrating this hypothesis will require studies with larger sample size.

## Abbreviations

HIV-1: human immunodeficiency virus type 1
VL: viral load
BSID-III: Bayley Scales of Infant Development III
PMTCT: prevention of mother-to-child transmission
ART: anti-retroviral therapy
CNS: central nervous system
HAND: HIV-associated neurocognitive disorders
HAD: HIV-associated dementia
VIF: variance inflation factors
sdNVP: single-dose nevirapine
